# Abscess of Gall-Bladder

**Published:** 1887-07

**Authors:** Henry L. Elsner

**Affiliations:** Syracuse, N. Y.


### *ABSCESS OF GALL-BLADDER.**

### CHOLECYSTOTOMY.—RECOVERY.

By HENRY L. ELSNER, M. D., Syracuse, N. Y.

On the 31st day of October, 1886, at about 9.30 P. M., I was hurriedly summoned to see W. S., aged 52 years, a furrier by

*Read before the Central New York Medical Association at Syracuse, N. Y., May 17, 1887.

occupation, a good liver, previously healthy, with the exception of constitutional syphilis thirty years ago, and two attacks of biliary colic, one twenty, the other ten years ago. The day upon which I was called was Sunday. He had rested, felt well, his bowels had moved regularly, and he had no symptoms which might lead him to anticipate the approach of disease. About 9 o'clock P. M., he was suddenly taken with a severe and acute pain, radiating from the right hypochondriac region backward, downward and upward. The pain was so severe that with every paroxysm of pain he had tonic and clonic contractions of the muscles of the extremities. He was unable to take the recumbent posture, but sat on a sofa, bent forward, his elbows resting upon his thighs, while his hands were pressing forcibly and continuously against the abdominal wall. There were moments when the pain was less severe, but at no time did he enjoy freedom from it. The severe pains recurred at intervals of from three to five minutes, with the convulsive movements attending. He vomited large quantities of a grass-green fluid, with abundant mucous. At times the free emesis seemed to relieve him for a few moments. The pain was so intense, the suffering so acute, added to the inability of the patient to lie down, that I was unable to make an examination of the abdominal organs or take his temperature. The pulse was 113 R. 20. A quarter of a grain of morphia was injected hypodermically, and repeated in half an hour, the first having failed to ameliorate the pain. In about twenty minutes after the second injection, the pains recurred at longer intervals, but the patient complained of great tenderness and soreness over the region of the gall-bladder. It was almost one o'clock A. M. before he could lie down, and then the pain recurred at intervals of one-half hour, with decreasing severity. He vomited several times during the night, and slept about one hour; by morning the acute pains had disappeared, the patient was able to walk out into the street contrary to my orders, and rode in a coupe to his place of business. These acute pains never returned during the subsequent history of the case.

November 1st—Able to sit up; unable to stand erect; the region of the gall-bladder seems swollen, abnormally prominent, very sensitive to pressure. He was ordered 10 grs. of blue mass, followed by an alkaline cathartic, also the phosphate of soda with a bitter tonic. T., P. and R. normal; no vomiting.

November 2d—Able to walk up-stairs to his room in the second story, but bent forward.

November 3d—Slightly jaundiced; some nausea; no vomiting; T. normal.

November 4th—Complains of pains when moving; no other noteworthy symptoms.

November 5th—T., P. and R. normal. Still walks bent forward; no pain, but tenderness as before. Physical examination negative.

From the 6th to the 13th there were no new developments, but the patient seemed to improve and to gain strength, though he did not walk as erect as formerly. On the 13th of November the glands in the right axilla were enlarged to the size of a goose egg, and the chain of glands extending to the elbow was also enlarged and tender. Tincture iodine was used locally, and the iodide of potassium internally.

December 8th—The swelling of the glands somewhat improved. A tumor can now be felt, occupying the right hypochondriac, right lumbar region, extending downward to the left, into the umbilical region, an inch beyond the umbilicus. It is hard, smooth, not painful to the touch, without fluctuation; the fingers can reach around its free border, and it is continuous with the liver dullness. The K. I. was continued.

December 11th—The axillary and bachial swellings have disappeared. The abdominal tumor has increased in size, while both extremities are œdematous, the right more than the left. From the 11th to the 29th of December the symptoms remained much the same; there was a continuous dragging sensation, referable to the right hypochondriac region; the extremities continuing œdematous, the right more than the left; the bowels confined except when relieved by Carlsbad salts. The pulse averaged 110°, T. 100, R. 20. Emaciation was progressive, appetite good, food well digested, and no vomiting.

December 29th—Dr. Didama saw the patient with me in consultation. The diagnosis was not made positive, though the doctor thought the tumor had an amyloid feel and might possibly be due to an amyloid and enlarged liver. The even enlargement with the previous history of constitutional syphilis seemed to strengthen this conclusion. The iodide was continued.

February 7th—The swelling can be distinctly outlined. It lies in the right hyp., lumbar and umbilical region, further downward and to the left than when last seen. Upon careful palpation, a slight fluctuation is perceptible to the right and about one-eighth of an inch from the umbilicus. This fluctuation extends upward towards the median line for about one inch. Around this line of fluctuation the tumor presents the usual hardness. Introducing a hypodermic, I withdraw a yellowish green fluid and pus. The pus looks healthy, the fluid contains abundant cholestrine plates, visible on microscopical examination. This aspiration, with previous history and microscopical examination, convinces me that we have a distended gall-bladder and abscess with which to deal. The patient growing more feeble and pressure symptoms developing, I decided to carefully, and under strict antiseptic precautions, cut into the swelling, following the line of fluctuation, introduce a drainage tube and wash out the cavity, as a last resort. The patient consented, and on the 9th of February I carefully dissected through the abdominal walls, and, following the line of fluctuation, caught the distended gall-bladder, incised it* and gave exit to one pint of pus and bile. The pus was laudable and thick, the bile of a yellowish green color. Air rushed in and out of the cavity freely ; the finger being introduced, could not feel the bottom of the gall-bladder. The cavity was washed out with a 1:5000 solution of chloride of mercury. A drainage tube was introduced and an iodoform dressing fastened over the opening by means of a roller bandage. This abscess cavity was washed out daily with

*The walls of the gall-bladder at point of incision were adherent to peritoneum.

a 1:300 solution of carbolic acid. At no time was there elevation of temperature; the sallow color of the patient disappeared; the tumor gradually became smaller; the œdema of the extremities was relieved; the draining and washing out was continued until there was no further discharge of pus, and by March 1st the tube could no longer be introduced.

On the 9th of March there was considerable redness and tenderness along the original line of incision. This was again opened and a drainage tube introduced, two ounces of pus escaping. The drainage tube was left until March 30th. For a few days there appeared to be a small fistulous opening, but this granulated and healed. Since April 3d the patient has been perfectly well, the gall-bladder has contracted, become adherent, or possibly obliterated. There is no sign of the original tumor, while physical examination fails to reveal the slightest abnormality.

